# Preparation of North American Type II PRRSV Infectious Clone Expressing Green Fluorescent Protein

**DOI:** 10.1155/2014/368581

**Published:** 2014-05-08

**Authors:** Liyue Wang, Kao Zhang, Hongyu Lin, Wenyan Li, Jiexia Wen, Jianlou Zhang, Yonghong Zhang, Xiujin Li, Fei Zhong

**Affiliations:** ^1^Laboratory of Molecular Virology and Immunology, College of Veterinary Medicine, Agricultural University of Hebei, Hebei Engineering and Technology Research Center of Veterinary Biotechnology, Baoding 071001, China; ^2^Section of Biology, Zhongyi Middle School of Xingtai, Hebei, China; ^3^Department of Biotechnology, College of Environmental and Chemical Engineering, Yanshan University, Qinhuangdao 066004, China

## Abstract

Porcine reproductive and respiratory syndrome virus (PRRSV) is still one of the most important infectious diseases threatening the swine industry. To construct North American type II PRRSV infectious clone containing green fluorescent protein (GFP) gene, we amplify gfp gene, flanked by PRRSV Nsp2 gene fragments upstream and downstream, using overlap PCR method from pcDNA-EF1-GFP plasmid and FL12 plasmid containing PRRSV infectious genome as the templates. The Nsp2 fragment-flanked gfp gene was inserted into Nsp2 gene of the FL12 plasmid by *Spe* I and *Xho* I sites to generate PRRSV infectious recombinant plasmid (FL12-GFP) containing gfp gene. The recombinant PRRSV expressing GFP (PRRSV-GFP) was rescued in baby hamster kidney-21 (BHK-21) cells by transfecting PRRSV mRNA synthesized *in vitro* and amplified in Marc-145 cells. The PRRSV-GFP infectivity and replication capacity were identified. Results showed that, by adopting overlap PCR strategy, the gfp gene was successfully inserted into and fused with PRRSV Nsp2 gene in the PRRSV infectious clone plasmid FL-12 to generate FL12-GFP plasmid. The recombinant PRRSV-GFP was generated through transfecting PRRSV mRNA in BHK-2 cells. Like its parental virus, the recombinant PRRSV-GFP maintains its infectivity to Marc-145 cells and porcine alveolar macrophages (PAMs). This study provides essential conditions for further investigation on PRRSV.

## 1. Introduction

Porcine reproductive and respiratory syndrome (PRRS), caused by PRRS virus (PRRSV), is one of the most serious infectious diseases in swine industry leading to huge economic loss worldwide [1, 2].

To investigate the interaction between virus and its host and to explore the viral pathogenetic mechanism at the molecular level, the viral infectious clone has been widely used since the viral genome could be conveniently modified on the infectious clone using molecular nucleic acid-based techniques to investigate virus-host interactions in viral infection, replication, and pathogenicity to the host. Therefore, many PRRSV infectious clones derived from different PRRSV strains have been constructed, such as PRRSV infectious clones derived from North American Type I and Type II PRRSV strains [3, 4], as well as the Chinese highly pathogenic PRRSV [5]. Infectious clone-based live-PRRSV vaccines have also been developed for prevention of PRRSV infection [6–9]. Although the vaccines showed a potential application prospect, the vaccinated pigs with this vaccine cannot be distinguished from pigs that have recovered from a natural infection. Therefore, many researches have been concerned with the genetically marked PRRSV vaccine. So the GFP-marked PRRSV infectious clone on North American type I (European Type I) was developed [3, 10], which would be valuable for assessing the effectiveness of PRRSV vaccine and investigating PRRSV-host interaction.

PRRSV is a small and enveloped virus containing a single, positive-stranded RNA genome and belongs to the family of Arteriviridae [11]. Based on its genomic sequence, PRRSV can be divided into European (type 1) and North American (type 2) genotypes [12, 13]. PRRSV genome is about 15 kb in size containing 9 open reading frames (ORFs). ORF1a and ORF1b at the 5′-end of the genome encode a polyprotein which was cleaved into 13 nonstructural proteins: Nsp1a, Nsp1b, and Nsp2 to Nsp12 [11–14]. ORF2-ORF7 at 3′-end of the genome encodes four membrane-associated glycoproteins (GP2, GP3, GP4, and GP5), two unglycosylated membrane proteins (E and M), and a nucleocapsid protein (N) [15–22].

The common strategy to prepare viral infectious clone containing genetic marker is to insert the exogenous marker gene into the viral genome or to substitute the fragment of viral genome with the marker gene. Therefore, selecting a proper site for marker modification is critical for successfully constructing the marked viral infectious clone. In view of the biological functions of PRRSV-encoded structural and nonstructural proteins in viral replication, pathogenicity, and immunogenicity, the Nsp2 protein was considered as a suitable candidate site for marker modification [3, 23, 24]. It has been proved that Nsp2 protein is the largest protein (1195 amino acids) in PRRSV nonstructural proteins, which contains multiple domains with many functions and plays a crucial role in viral replication [25–27]. The Nsp2 is also a variable protein which can tolerate large deletions and insertions [28] within the central region of the Nsp2 protein [28–32]. As its cysteine protease domain which is essential for Nsp2/3 cleavage [25–27] is located in the N terminus, the active centre of which is located in the 181–323 amino acid (it counts for 12% of the NSP2 gene) [33], therefore, the deletion and insertion in the central region of the Nsp2 do not significantly interfere with Nsp2 functions. For instance, Fang et al. have inserted gfp gene into NSP2-encoded region of North American type I (SD01-08 strain) infectious clone at the position between AA_733_ and AA_744_ downstream of protease core sequence to generate the North American type I GFP-PRRSV infectious clone [3].

North American type II PRRSV, like type I, is still the widespread genotype of PRRSV in many countries in the world, especially in Asian countries. To explore the virus-host interaction and develop the infectious clone-based PRRSV vaccine, the genetically-marked infectious clone of North American Type II PRRSV is needed. In this study, we prepared the GFP-marked infectious clone for North American Type II PRRSV by inserting gfp gene into Nsp2 encoding region of North American Type II PRRSV (NVSL 97-7895 strain) infectious clone, which might provide one of the favorable conditions for further investigation on North American Type II PRRSV.

## 2. Materials and Methods

### 2.1. Plasmids, Cell Lines, and Bacteria

PRRSV infectious clone plasmid FL12 derived from North American Type II PRRSV (NVSL 97-78950 strain) was a generous gift provided by Asit K. Pattnaik from the University of Nebraska. The pcDNA-EF1-GFP plasmid containing gfp gene was from Shanghai Benefit Biotechnology. Baby hamster kidney-21 (BHK-21) cells, Marc-145 cells, and human embryonic kidney 293T (HEK293T) were from China Center for Type Culture Collection. All of the cells were cultured in DMEM medium supplemented with 10% (v/v) FBS, 100 U/mL penicillin, 100 *μ*g/mL streptomycin, 2 mM L-glutamine at 37°C in 5% CO_2_ incubator. Porcine alveolar macrophages were isolated from lungs of healthy piglet and cultured in RPMI-1640 medium supplemented with the same components as above.* Escherichia coli* DH5*α* were from our laboratory.

### 2.2. Reagents

mMESSAGE mMACHINE High Yield Capped RNA Transcription kit was from Ambion (Austin, TX, USA). TransMessenger Transfection Reagent, RPMI-1640 medium, X-VIVO-15 serum-free medium, L-glutamine, and fetal calf serum were from Life Technologies Gibco/BRL division (Grand Island, NY, USA). Yeast poly(A) polymerase and* Acl* I enzyme were from United States Biochemical (Cleveland, OH, USA). Other restriction enzymes, T4 DNA ligase, and PureYield Plasmid Miniprep System were from Promega (Madison, WI, USA). DNA marker DL2000 was from Takara Biotechnology (Dalian) (Dalian, China). The other reagents were analytical grade chemicals from domestic and international companies.

### 2.3. Amplification of gfp Gene Fused with Nsp2 Fragments at Both Up- and Downstream

**Figure 1:**
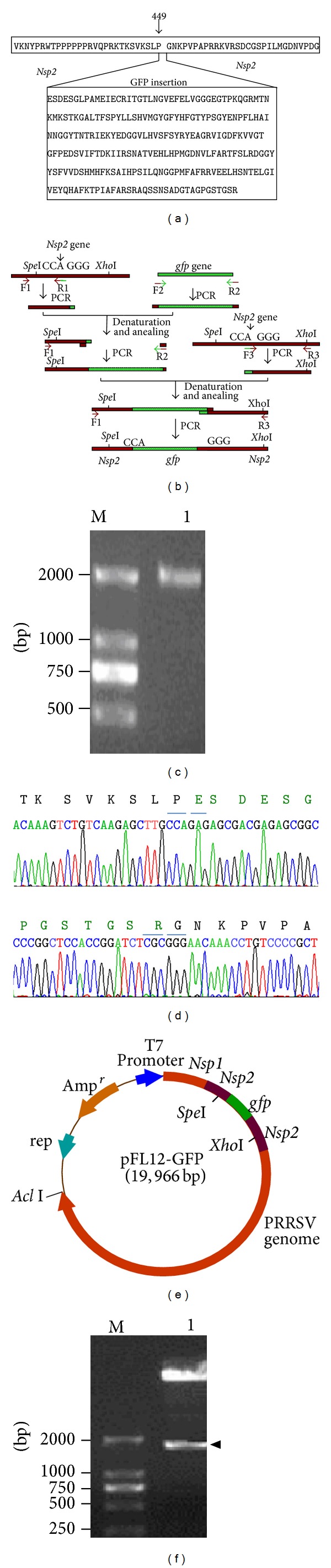
Amplification of the Nsp2-GFP fragment by overlap PCR and the construction of PRRSV infectious clone pFL12-GFP. (a) Inserting site of gfp gene between proline and glycine of Nsp2 encoding region. (b) Amplifying strategy of Nsp2-GFP fragment by overlap PCR. F1~F3 and R1~R3 present primers. (c) PCR product of Nsp2-GFP gene amplified by overlap PCR. M, DL 2000 DNA Marker; Lane 1, Nsp2-GFP gene fragment. (d) The sequences of Nsp2-gfp and gfp-Nsp2 fusion regions. (e) Structure of pFL12-GFP plasmid. The Nsp-GFP gene fragment was inserted into Nsp2 encoding region at* Spe* I and* Xho* I sites, in which the gfp gene was fused with Nsp2 at 5′ and 3′ terminus and controlled by T7 promoter. (f) Restriction analysis of the pFL12-GFP plasmid. M, DL 2000 DNA Marker; Lane 1, digested fragments of the pFL12-GFP plasmid by* Spe* I and* Xho* I.

The overlap PCR was used for amplification of the Nsp2-fused gfp gene. Based on PRRSV Nsp2 sequence in pFL12 plasmid and gfp sequence in pcDNA-EF1-GFP plasmid, 3 pairs of primers were designed (Table 1) for amplifying overlapped 3 fragments. R1 primer is overlapped with F2 primer, and R2 primer is overlapped with F3 primer. Then, the Nsp2-fused GFP fragment (1860 bp) was generated by overlap PCR based on the designed strategy (see Figure 1(b)).

PCR thermal cycling conditions for amplifying the different fragments were as follows: initial denaturation at 94°C for 5 min and then 30 cycles of denaturation at 94°C for 1 min, annealing at the temperature (Table 2) for 1 min, and extension at 72°C for the different time (Table 2) and the final extension at 72°C for 10 min.

### 2.4. Construction of PRRSV Infectious Clone Plasmid Containing gfp Gene

The overlap PCR-amplified Nsp2-GFP fragments (1860 bp) were purified and cloned into pMD19-T vector; after being identified by sequencing, the Nsp2-GFP fragment was subcloned into FL12 plasmid by Spe I and Xho I sites, where the fragment in Nsp2 gene between Spe I and Xho I sites in FL12 was substituted with Nsp2-GFP fragment to generate the recombinant PRRSV infectious clone plasmid containing gfp gene fused with Nsp2, named FL12-GFP plasmid. The recombinant plasmid was identified by restriction analysis.

### 2.5. Synthesis of PRRSV mRNA* In Vitro*

PRRSV mRNA was synthesized* in vitro* as described previously [34]. Briefly, FL12-GFP plasmids were prepared with PureYield Plasmid Miniprep System kit and linearized by* Acl* I digestion at the site downstream of PRRSV genome. The linearized FL-12-GFP DNA was purified through protease K/SDS digestion, phenol/chloroform extraction, and ethanol precipitation. The 5′-end capped GFP-PRRSV mRNA was transcribed as previously described [34] by T7 RNA polymerase* in vitro* with mMESSAGE mMACHINE Transcription kit using linearized plasmid as a template. The poly(A) tail was added at the 3′-end of the capped GFP-PRRSV mRNA by yeast poly(A) polymerase to generate 5′-end capped and 3′-end tailed GFP-PRRSV mRNA. The mRNA size and the concentration were measured by denaturing agarose gel electrophoresis and NanoDrop 2000 (Thermo Science) spectrophotometry, respectively.

### 2.6. GFP-PRRSV mRNA Transfection and Recombinant GFP-PRRSV Generation

BHK-21 cells were seeded in 6-well plate the day before transfection. When the cell density reached 85%~90% confluence, the GFP-PRRSV mRNA (3 *μ*g) was transfected into BHK-21 cells using TransMessenger Transfection Reagents according to the manufacturer's instructions. After incubation at 37°C, 5% CO_2_ for 48 h, the transfected cells were broken by repeated freezing/thawing cycles; the supernatant separated by centrifuge was harvested and used to infect freshly seeded BHK-21 cells. The above infection/harvest process (blind passage) was repeated for 3-4 times for viral packaging. After repeated cycles, the harvested supernatant was used to infect Marc-145 cells for GFP-PRRSV amplification. The GFP expression was directly detected at 24–72 h after infection under a fluorescence microscope.

### 2.7. GFP-PRRSV Amplification and Viral Titer Determination

GFP-PRRSV was amplified in Marc-145 cells and PRRSV titer was determined by 50% tissue culture infective dose (TCID_50_).

## 3. Results

### 3.1. The Construction of PRRSV Infectious Clone Plasmid Containing gfp Gene

To construct GFP-PRRSV infectious clone, the inserting site for gfp gene should be selected properly so as to make gfp gene express efficiently and the GFP-PRRSV infection and replication in the host cells should not be significantly affected upon gfp gene insertion. As mentioned above, PRRSV NSP2 is a variable nonstructural protein in which the inserted exogenous DNA segment downstream Nsp2 protease core sequence would not seriously affect PRRSV replication [11, 13]. Therefore we inserted exogenous gfp gene at CCA_1347_ ↓ G_1348_GG position downstream protease core sequence, corresponding to the position between Pro_449_ and Gly_450_ (Figure 1(a)), and the open reading frame of Nsp2 downstream gfp gene did not change since the gfp gene was fused with Nsp2 protein. The specific* Spe *I site upstream and* Xho* I site downstream gfp inserting position was also considered during the inserting site determination.

After determining the inserting site, based on the amplification strategy (Figure 1(b)), the Nsp2-GFP gene was amplified by overlap PCR using PRRSV infectious clone plasmid FL12 and gfp gene-contained plasmid pcDNA-EF1-GFP as the templates. About 1900 bp DNA fragment was amplified (Figure 1(c)) as expected. The amplified Nsp2-GFP gene was cloned into pMD19-T vector and sequenced by Sangon Biotech (Shanghai), showing that the sequence of Nsp2-GFP DNA amplified was consistent with the templates; Figure 1(d) showed the sequence of Nsp2-gfp and gfp-Nsp2 fusion sites. The Nsp2-GFP DNA was then subcloned into FL12 plasmid by* Spe* I and* Xho* I sites to construct gfp gene-contained PRRSV infectious clone plasmid FL12-GFP (Figure 1(d)). Restriction analysis showed that about 1700 bp expected DNA fragment (Figure 1(d)) was cut out from FL12-GFP plasmid by double digestionwith* Spe* I and* Xho* I, suggesting that Nsp2-GFP gene has been inserted into the expected site of Nsp2 gene.

### 3.2. Synthesis of GFP-PRRSV mRNA* In Vitro* and Generation of Recombinant PRRSV Virus Expressing gfp Gene

**Figure 2:**
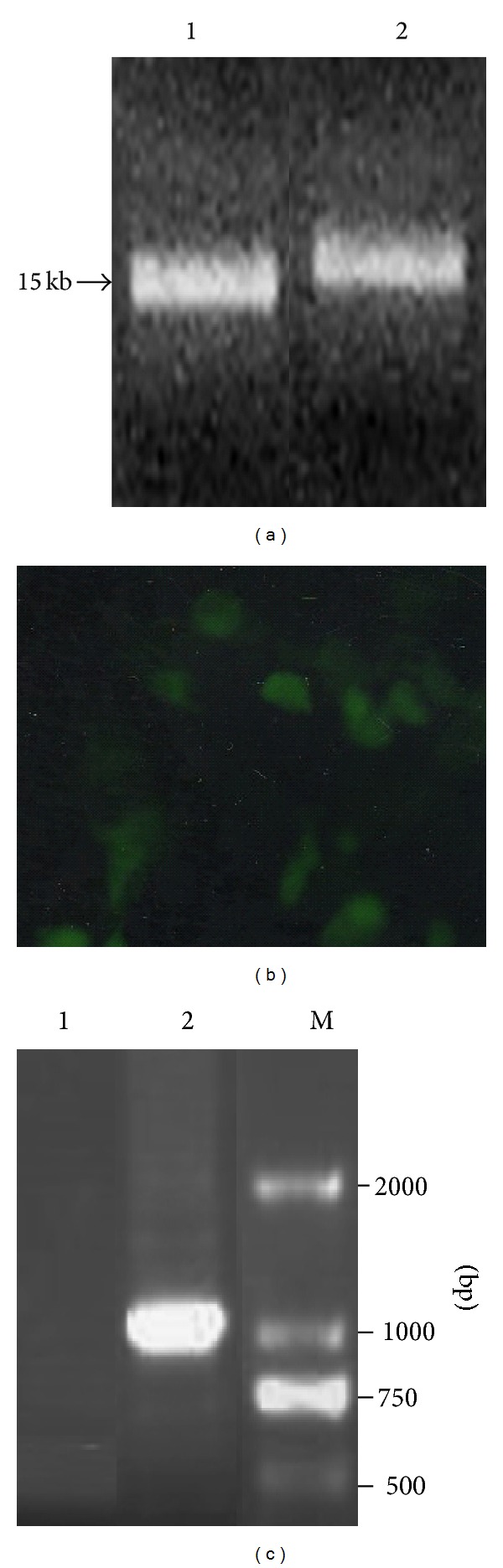
Synthesis of PRRSV-GFP mRNA* in vitro* and generation of the recombinant PRRSV containing gfp gene. (a) Electrophoresis of PRRSV-GFP mRNA (Lane 1) and PRRSV-GFP-Poly(A) mRNA (Lane 2) on 0.5% denaturing agarose gel, synthesized* in vitro* by T7 RNA polymerase using mMESSAGE mMACHINE mRNA Transcription kit. (b) GFP expression in Marc-145 cells infected for 72 h with the supernatant of the 3rd time of blind passage in BHK-21 cells originally transfected with PRRSV-GFP mRNA. (c) Identification of PRRSV expressing GFP by amplifying specific fragment by RT-PCR from total RNA from Marc-145 cells infected with PRRSV (Lane 1) and GFP-PRRSV (Lane 2). About 1100 bp fragment was amplified by RT-PCR with a pair of primers (F1 and R2).

GFP-PRRSV generation was performed as described previously [3, 10, 35]. The 5′-capped GFP-PRRSV mRNA (Figure 2(a)) was first transcripted by T7 DNA polymerase* in vitro* with the transcription kit using* Acl* I-linearized FL12-GFP plasmid as a template (there is a T7 promoter upstream PRRSV genome in FL12 plasmid). The 5′-capped mRNA was tailed in 3′-end catalyzed by poly(A) polymerase. The synthesized 5′-capped and 5′-capped/3′-tailed GFP-PRRSV mRNA size was checked by denaturing agarose gel electrophoresis (0.5%) and the mRNA concentration was measured by NanoDrop Spectrophotometer. Figure 2(a) showed the 5′-capped and its 3′-tailed GFP-PRRSV mRNA sizes, which were consistent with the expected sizes. The 5′-capped and 3′-tailed GFP-PRRSV mRNA was transfected into BHK-21 cells followed by 3 generation blind passages in the cells for viral packaging. Finally, the packaged virus in the supernatant was used to infect Marc-145 cells for viral amplification. At 48 h after infection, GFP expression in some cells was detected in the Marc-145 cells and, at 72 h, the GFP expression in the cells became more visible (Figure 2(b)), which illustrates that the generated GFP-PRRSV can replicate in the host Marc-145 cells.

To further verify that the generated recombinant virus is the GFP-PRRSV, we used RT-PCR to amplify the specific fragment (Nsp2-gfp DNA segment, about 1100 bp in length) from GFP-PRRSV-infected cells with F1 and R2 primers mentioned above. As shown in Figure 2(c), about 1100 bp DNA fragment was amplified from GFP-PRRSV-infected Marc-145 cells, but not from PRRSV-infected cells, indicating that the GFP-PRRSV has been successfully constructed in this study.

### 3.3. Recombinant GFP-PRRSV Could Replicate in Host Cells

**Figure 3:**
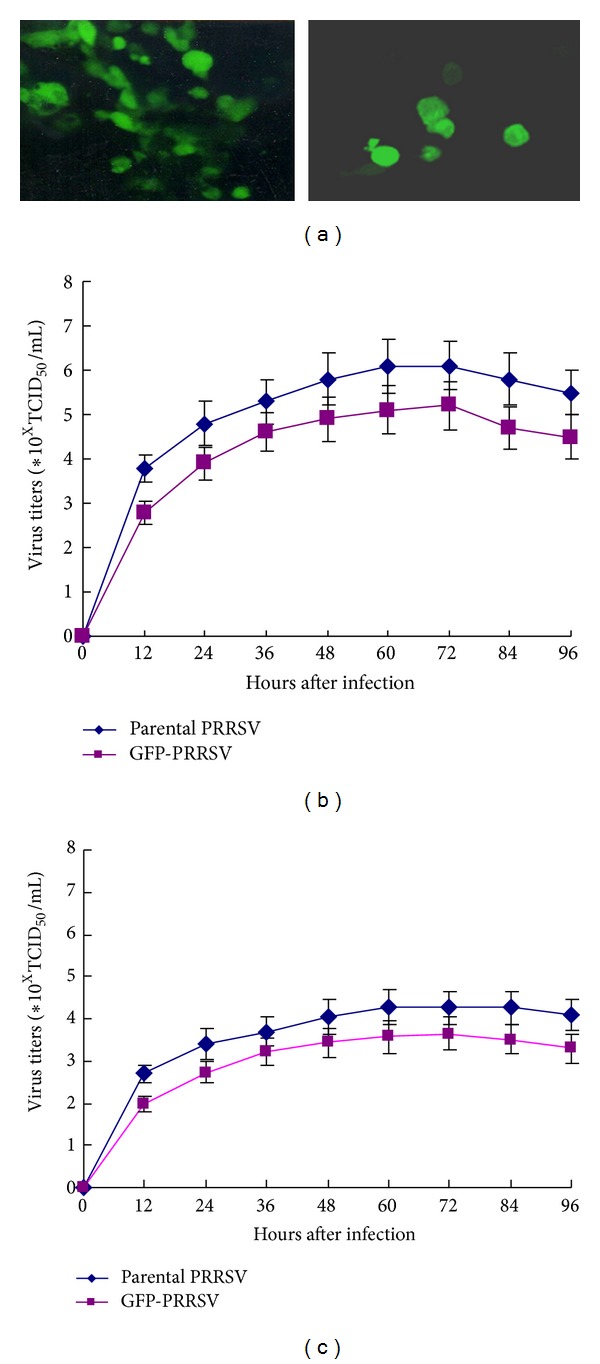
Replication capacity of GFP-PRRSV in Marc-145 cells and PAMs. (a) GFP expression in Marc-145 cells and PAMs infected by PRRSV-GFP virus at 48 h, respectively. (b) PRRSV and GFP-PRRSV titers in Marc-145 cells at different times after infection. (c) PRRSV and GFP-PRRSV titers in PAMs at different times after infection.

As illustrated above, GFP-PRRSV regenerated by GFP-PRRSV infectious clone could replicate in Marc-145 cells. To investigate whether the GFP-PRRSV could infect and replicate in porcine alveolar macrophages and to compare the replication capacity in those two cells* in vitro*, we infected Marc-145 cells and PAMs with GFP-PRRSV and its parental PRRSV and measured the viral titers in different cells at different infection periods (12, 24, 36, 48, 60, 72, and 84 h). The viral replication capacities of GFP-PRRSV and its parental PRRSV were estimated based on the viral titers in Marc-145 and PAMs (Figure 3).

The results in Figure 3(a) showed that, after GFP-PRRSV infection, the percentage of the GFP-expressed Marc-145 cells was significantly higher than that of the GFP-expressed PAMs at 48 h after infection, suggesting that the infectivity of GFP-PRRSV was more efficient in Marc-145 cells than in PAMs. It can be seen from Figure 3 that the replication ability of GFP-PRRSV, similar to its parental PRRSV, was higher in Marc-145 cells than that in PAMs, and the GFP-PRRSV replication is slower than that of its parental PRRSV in either Marc-145 cells or PAMs, indicating that the gfp gene insertion likely decreases PRRSV replication in the host cells. However, the low-replication of GFP-PRRSV would not obstruct its application in future studies.

## 4. Discussion

In this study, we constructed PRRSV infectious clone containing gfp gene on its parental infectious clone derived from America PRRSV type II genotype using overlap PCR and other molecular techniques by inserting gfp gene into the Nsp2 gene of the PRRSV genome. Like the parental PRRSV, the GFP-PRRSV rescued from the GFP-PRRSV infectious clone can infect Marc-145 cells and PAMs, replicate in those two cells, and mediate inserted gfp gene expression in the cells. The GFP-PRRSV infectious clone constructed in this study provides not only the favorable condition for investigating the pathogen biology of North America PRRSV type II but also the experimental data for further developing the genetically marked PRRSV vaccine.

To insert exogenous gene, such as genetic marker genes (gfp and lacZ) or other functional genes, into viral genome for preparation of recombinant virus, the insertion site in viral genome should be taken into consideration. There is a wide variety of virus types with different sizes and different functions of genome either coding region or noncoding region, playing different roles in virus replication and pathogenicity to the host. Therefore, the site for exogenous gene inserting should be comprehensively considered based on viral genomic characteristics, and the selected inserting site should be finally determined by experiment. Generally, the basic requirement for the insertion site selection is that the inserted gene should not significantly influence the virus replication. In general, the replication nonessential regions of the viral genome are considered as the candidate regions for exogenous gene insertion or substitution. For instance, human adenovirus vector with deletion of the nonessential E3 region can provide enough room for the exogenous gene insertion, since, without the deletion, the large gene insertion should seriously affect virus packaging [36]. If the replication-essential or nonessential region has not been identified, the highly variable regions either coding regions or noncoding regions, especially the region where the large DNA or RNA fragment insertion or deletion often takes place, were considered as the candidate inserting sites because the virus can tolerate the mutation in these regions [37]. Based on the previously reported strategies [3, 33], PRRSV Nsp2 gene chosen as the site for gfp gene insertion is also due to considering that Nsp2 gene varies greatly in different PRRSV strains. It has been reported that 30-amino-acid deletion in Nsp2 region did not affect PRRSV virulence [38]. Therefore, inserting exogenous gene into Nsp2 gene at the proper site is feasible.

The coding regions for viral nonstructural proteins, especially for the proteins with multistructural domains, are commonly selected to be the candidate inserting sites for exogenous genes. The exogenous genes are often inserted on the border between two structural domains so as to avoid the serious influence on the structure and functions of the original viral proteins. PRRSV Nsp2 is a protein with multistructural domains and its protease domain is located between AA_181_ and AA_323_ (called core sequence) [3]. Our result showed that inserting gfp gene downstream of Nsp2 protease core sequence did not affect PRRSV replication seriously.

The strategy of inserting exogenous gene into viral nonstructural protein coding region and fusing the gene with it as well could avoid adding promoter at the upstream of exogenous gene and poly(A) at the downstream. Meanwhile, since the inserted gene is fused with viral nonstructural protein gene, the open reading frame of the viral coding region downstream of the inserted gene should not be changed after the insertion; the viral gene-encoded protein sequence downstream of the inserted site should not be interrupted.

## Figures and Tables

**Table 1:** Primers for amplifying gfp gene fused with PRRSV Nsp2 gene sequence.

Primers	Sequence of primers (5′ → 3′)	Sizes/bp	Location in the plasmids
F1	CTACTATCCT GCACAAGGTG	355	2351~2370 (FL12)
R1	CAGGCCGCTCTCGTCGCTCTCTGGCAAGCTCTTGACAGACTTTG		9854~9875 (pCDNA-EF1-GFP) 2662~2684 (FL12)

F2	CAAAGTCTGTCAAGAGCTTGCCAGAGAGCGACGAGAGCGGCCTG	800	2662~2684 (FL12) 9854~9875 (pCDNA-EF1-GFP)
R2	GCGGGGACAGGTTTGTTCCCGCGAGATCCGGTGGAGCC		2684~2704 (FL12) 10589~10607 (pCDNA-EF1-GFP)

F3	GGCTCCACCGGATCTCGCGGGAACAAACCTGTCCCCGC	840	10589~10607 (pCDNA-EF1-GFP) 2684~2704 (FL12)
R3	GGTGTCTCGAGTATCATCTTTG		3481~3502 (FL12)

**Table 2:** Tm and extension time for amplifying the corresponding segment.

PCR fragments	Annealing temperature (°C)	Extension time (S)
Frag-1	56	30
Frag-2	58	60
Frag-3	58	60
Frag-4	58	90
Nsp2-GFP frag	60	120
